# Machine Learning-Driven Prediction of Spatiotemporal Dynamics of Active Nuclei During *Drosophila* Embryogenesis

**DOI:** 10.3390/ijms262110338

**Published:** 2025-10-23

**Authors:** Parisa Boodaghi Malidarreh, Priyanshi Borad, Biraaj Rout, Anna Makridou, Shiva Abbasi, Mohammad Sadegh Nasr, Jillur Rahman Saurav, Kelli D. Fenelon, Jai Prakash Veerla, Jacob M. Luber, Theodora Koromila

**Affiliations:** 1 Department of Computer Science and Engineering, University of Texas at Arlington, Arlington, TX 76019, USA; pxb7330@mavs.uta.edu (P.B.M.); bxr1886@mavs.uta.edu (B.R.); mohammadsadegh.nasr@mavs.uta.edu (M.S.N.); mxs2361@mavs.uta.edu (J.R.S.); jxv6663@mavs.uta.edu (J.P.V.); 2 Multi-Interprofessional Center for Health Informatics, University of Texas at Arlington, Arlington, TX 76019, USA; 3 Department of Biology, University of Texas at Arlington, Arlington, TX 76019, USA; phb9243@mavs.uta.edu (P.B.); sxa1511@mavs.uta.edu (S.A.); 4 School of Biology, Aristotle University of Thessaloniki, 54124 Thessaloniki, Greece; annmak4@gmail.com; 5 Department of Psychiatry, University of Texas Southwestern Medical Center, Dallas, TX 75390, USA; kelli.fenelon@utsouthwestern.edu; 6 Department of Bioengineering, University of Texas at Arlington, Arlington, TX 76019, USA

**Keywords:** XGBoost, regression, *Drosophila* embryo, *sog*, *Su*(*H*), Ripley’s K-function, MS2.MCP live imaging system

## Abstract

In this study, we apply machine learning to model the spatiotemporal dynamics of gene expression during early *Drosophila* embryogenesis. By optimizing model architecture, feature selection, and spatial grid resolution, we developed a predictive pipeline capable of accurately classifying active nuclei and forecasting their future distribution in time. We evaluated the model on two reporter constructs for the *short gastrulation* (*sog*) gene, *sogD* and *sogD_∆Su*(*H*), allowing us to assess its performance across distinct genetic contexts. The model achieved high accuracy on the wild-type *sogD* dataset, particularly along the dorsal–ventral (DV) axis during nuclear cycle 14 (NC14), and accurately predicted expression in the central regions of both wild-type and Suppressor of Hairless (Su(H)) mutant enhancers, *sogD_∆Su*(*H*). Bootstrap analysis confirmed that the model performed better in the central region than at the edges, where prediction accuracy dropped. Our previous work showed that Su(H) can act both as a repressor at the borders and as a stabilizer of transcriptional bursts in the center of the *sog* expression domain. This dual function is not unique to Su(H); other broadly expressed transcription factors also exhibit context-dependent regulatory roles, functioning as activators in some regions and repressors in others. These results highlight the importance of spatial context in transcriptional regulation and demonstrate the ability of machine learning to capture such nuanced behavior. Looking ahead, incorporating mechanistic features such as transcriptional bursting parameters into predictive models could enable simulations that forecast not just where genes are expressed but also how their dynamics unfold over time. This form of in silico enhancer mutagenesis would make it possible to predict the effects of specific binding site changes on both spatial expression patterns and underlying transcriptional activity, offering a powerful framework for studying cis-regulatory logic and modeling early developmental processes across diverse genetic backgrounds.

## 1. Introduction

Recent technological advances have made it possible to capture high resolution images from embryogenesis processes that help researchers to study gene expression patterns [1,2]. One of the major challenges of the modern genomics era is to better understand how gene expression is regulated to support spatiotemporal outputs that change over the course of development. It is known that multiple, transiently acting enhancers function sequentially to regulate dynamic changes in gene expression outputs [2,3,4,5], whereas other genes are controlled by enhancers that act over a longer period and support changing spatial outputs over time. The early *Drosophila* embryo has served as a paradigm for how enhancers and transcription factors control gene patterning. For example, the gene *short gastrulation* (*sog*) is a critical early zygotic target in *Drosophila* embryogenesis, activated by a combination of transcription factors that establish spatial gene expression patterns. The expression of the *sog* gene is driven by at least two co-acting enhancers, *sog_distal* and *sog_intronic*, that support temporally dynamic expression. Its activation is initiated by the pioneer factor Zelda (Zld), which opens chromatin to facilitate binding by other regulators. Broadly expressed activators such as Dorsal (Dl) and Twist (Twi) further drive *sog* transcription in ventrolateral regions of the embryo. At the same time, repressors like Suppressor of Hairless (Su(H) restrict *sog* expression to ensure proper boundary formation, contributing to precise patterning during early development.

Live imaging experiments enable the potential to analyze gene expression dynamics with increased temporal resolution and linear quantification [6]. However, genetic and live imaging techniques have outpaced analysis approaches to harvest the bountiful information contained within real time movies of transcriptional dynamics with modern methods confined to static parameter cell and transcript tracking methods [1,7,8]. In this study, we developed a quantitative approach to measure the spatiotemporal outputs of *sog_distal* enhancer-driven expression, demonstrating that gene expression is complex and dynamic [9]. Using transgenic fly lines, we conducted live imaging to visualize the RNA nascent transcripts associated with MS2 [10,11] stem-loop reporter sequence binding MCP-GFP, enabling dynamic tracking of RNA localization and expression in real time. In this study, we extend these insights by applying machine learning to examine, for the first time, a repressor’s dual activities on the same enhancer. Manno et al. [12] introduced the concept of RNA velocity, which is defined as the time derivative of gene expression, offering a novel approach for inferring dynamic changes in gene activity over time. T. Dayao et al. [13] employed Ripley’s K-function to capture spatial gene expression outputs, which inspired the development of our proposed pipeline. Using this approach, we developed a feature extraction method and analysis pipeline capable of predicting the future distribution of nuclei expressing the *sog* gene. This approach enables in silico enhancer mutagenesis, allowing predictions of how specific binding site alterations affect transcriptional dynamics prior to experimental validation. Notably, it also allows, for the first time, the prediction of differential regulatory activities of transcription factors within the same gene. Our previous work showed that Su(H) activity can exert opposite roles depending on spatial context, stabilizing transcriptional bursts at the center of the *sog* expression domain while restricting expression at the borders [14].

## 2. Results

### 2.1. Analysis of Transcription Factor Binding and Chromatin Accessibility at the sog_distal Enhancer

**Figure 1:**
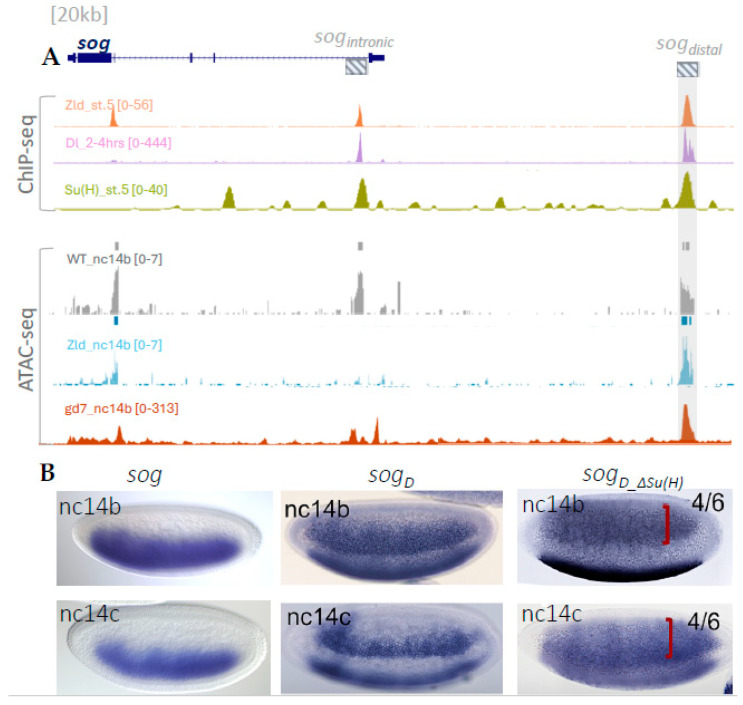
Regulation of *sog_distal* by TFs and chromatin accessibility during early Drosophila development. (**A**) Brennan et al. (2023 [15], *Developmental Cell*) reported that chromatin accessibility and target gene activation do not always correlate. This observation is consistent with our ATAC-seq data for gd7, which indicate that although Dorsal regulates *sog* expression, it does not affect chromatin accessibility—chromatin remains open regardless of Dorsal activity. For *sog_distal,* our data suggest distinct regulatory behavior. The *sog_distal* enhancer consistently regulates chromatin accessibility and contributes to *sog* expression. (**B**) The first two images show the endogenous *sog* expression from BDGP data base. The images in Panel B adapted from Koromila et al. (2019 [1], *Cell Reports*) show embryos at stages NC14b and NC14c, which were stained by in situ hybridization intronic yellow riboprobe to assay reporter expression supported by constructs *sog_Distal* and *sog_D__ΔSu(H).* Red brackets show the expanded *sog_Distal* expression pattern associated with mutant constructs (*n* = 6). In this and subsequent panels, lateral or ventrolateral views of embryos are shown with their anterior side to the left and their dorsal side up, unless otherwise noted.

ChIP-seq meta-analysis confirmed binding of Zelda (Zld), Dorsal (Dl), and Su(H) at the *sog* locus, with strong enrichment at the s*og_distal* enhancer (Figure 1A). ATAC-seq profiles further revealed that chromatin accessibility at this region remains open, indicating that accessibility is maintained independent of Dorsal or Zelda activity (Figure 1A). This supports previous findings that chromatin accessibility and target gene activation are not always correlated [15]. Within the *sog_distal* enhancer sequence, Su(H) binding sites have been identified [1]. Mutation of Su(H) sites in *sogD_*Δ*Su*(*H*) was previously shown to expand the reporter expression domain at NC14, confirming the role of Su(H) as a repressor at the borders [1,14]. Consistent with this, in situ hybridization assays confirm that loss of Su(H) input leads to an expansion of *sog* expression at NC14b–c (Figure 1B), in agreement with previous findings. Notably, Fenelon et al. [16] demonstrated that Su(H) has differential regulatory roles depending on the spatial context, acting distinctly at the borders versus the center of the expression domain. Together, these results indicate that Su(H) regulates *sog_distal* in context-dependent ways.

### 2.2. Comprehensive Analysis of Super-Resolution Live Movies

To capture the spatiotemporal dynamics of gene expression outputs, we developed an image processing approach to collect detailed information in both time and space by capturing the lateral half of the embryos [17]. With this qualified imaging dataset, our goal was to predict the distribution of active nuclei in each stage of embryonic development as the blastula transitions into gastrulation. In this work, we further explore machine learning models for the prediction of the differential regulatory activities. As outlined in the methodology section, during the feature extraction phase, square grids were applied to images, and the number of active cells within each grid was predicted. The key challenge was selecting the optimal grid size to enhance performance on test data.

**Figure 2:**
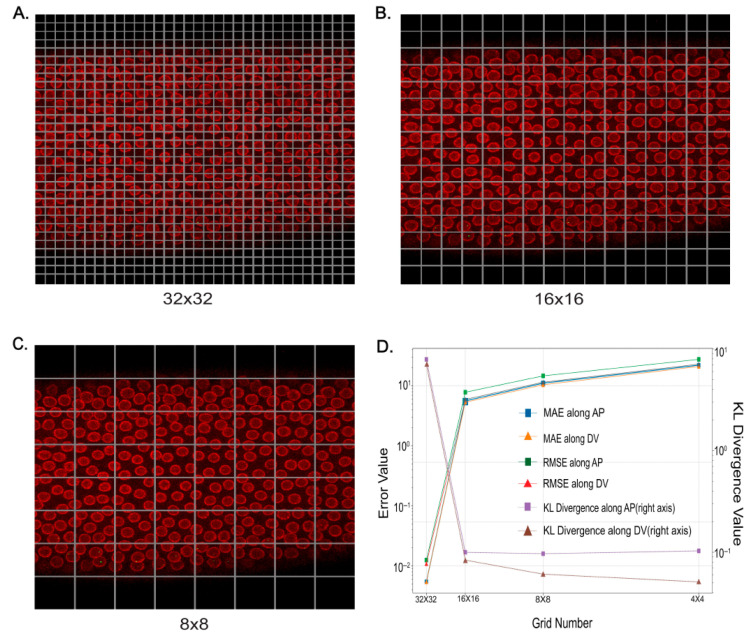
Optimization of live imaging analysis. (**A**–**C**) Depictions of one frame of a NC13 embryo with three distinct grid configurations, labeled A, B, and C, corresponding to grid sizes of 32 × 32, 26 × 26, and 8 × 8, respectively. (**D**) Error plot associated with each grid configuration (**A**–**C**), facilitating the identification of the optimal grid size based on the lowest error value.

Consequently, we replicated the entire process of pre-processing and feature extraction for four distinct grid sizes: 250, 125, 62.5, and 31.25 (where the grid size of ‘*n*’ indicates the division of the entire image into n* n squares). We used three different metrics to calculate the model performance on test data for different grid sizes, which are rmse (root mean squared error), mae (mean absolute error), and Kullback–Leibler (KL) Divergence. Figure 2 shows the experiment for different grid sizes.

Our analysis revealed the same increasing trend in both rmse and mae as the grid size increases from 31.25 to 250 which indicated that a smaller grid size corresponds to a lower error. KL Divergence, which we also utilized as a metric, measures how one probability distribution diverges from a second one. Thus, the smaller value for it shows that two distributions are closer to each other. We used this criterion to see how well the pipeline can capture trends in the active cell distribution. The KL Divergence for these four different grid sizes showed different trends. Increasing the grid size from 31.25 to 250 yielded a decrease in the KL Divergence. We had two options; the first one was to select 31.25 based on its lower rmse and mae. However, the problem was the average size of the cell was approximately 36. When we set the grid size to 31.25, each grid contained only one cell, effectively turning the function into a binary classification of each grid as either active or inactive — which was not our intended purpose. Another option was to select the optimal grid size based on KL Divergence, which, finally, we did, selecting the grid size of 62.5 over 31.25. The decision of selecting 62.5 over 125.0 despite 125 having a lower KL Divergence is attributed to the computational constraints of calculating Ripley’s K-function for larger grid sizes in our set-up.

In a subsequent experiment, we conducted an ablation study to discern the relative importance of features, identifying those deemed crucial for inclusion in the final release and those that could be omitted. Table 1 indicates the performance of different combinations of featuresThe features in the first row, including Ripley’s K-function and *n*, were the most important features that we used for training and testing the pipeline. All mae values were validated using K-fold cross-validation to reduce random variability.

**Figure 3:**
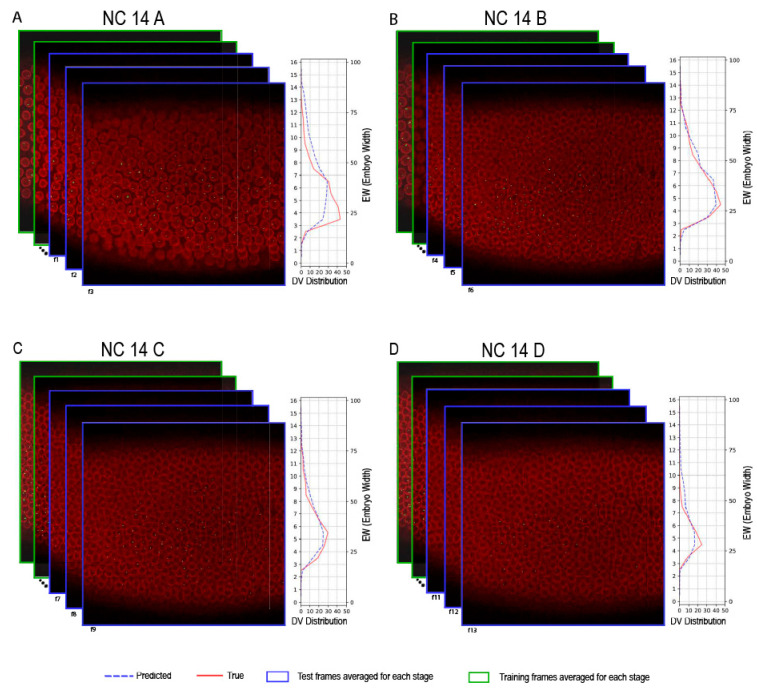
Processed live imaging data of *sogD* expression before gastrulation (NC14). The distribution of active cells achieving the best accuracy, based on mae values, is shown for the four stages of NC14 (**A**–**D**). In panels A–D, green rectangles indicate the frames from the previous stage used to predict the blue frames of the current stage. The features from the previous stage frames were averaged to predict the average number of active cells in each grid for the current stage. For each stage, the right-hand plot illustrates the predicted and actual distribution of active cells along the DV axis, represented by dashed blue and red lines, respectively. In these plots, the grid numbers along the DV axis are shown from 0 to 16, the average number of active cells per grid is displayed from 0 to 50, and the embryo width along the DV axis spans from 0 to 100.

To visualize the performance of the pipeline with selected features and parameters, we tested the pre-trained model on a test dataset. Figure 3 shows the active cell distribution for the best prediction based on the average mae values.

### 2.3. Comparative Evaluation of sogD and sogD Su(H)

As we had six videos for *sogD*_∆*Su*(*H*) and seven for *sogD*, we randomly selected three videos from each group for training and one for testing. Then, we averaged the AP mae, DV mae, and mean mae for the *sogD* and *sogD*_∆*Su*(*H*) experiments and calculated the difference between *sogD* and *sogD*_∆*Su*(*H*) for each of these metrics, and the results were 0.210, 1.511, and 0.86, respectively. We also used cross-validation to avoid overfitting. These results show that there was a difference between the performance of our pipeline on *sogD*_∆*Su*(*H*) and *sogD* in AP mean, mean mae, and DV mean. In other words, our method worked better in predicting along the AP axis, the mean of AP, and DV on the *sogD* data compared to the *sogD*_∆*Su*(*H*) data. To substantiate this assertion, we conducted two additional experiments:

First, we leveraged mixed-effects modeling, which can account for both fixed effects (like the group *sogD* or *sogD*_∆*Su*(*H*)) and random effects (like variation within videos and stages). The mixed-effects model can help in understanding the influence of these fixed and random effects on our dependent variables such as DV mae, AP mae, and mean mae. The goal is to understand whether there is a significant difference in any metrics between the *sogD*_∆*Su*(*H*) and *sogD* groups which account for the variability introduced by different stages. The *sogD* had, on average, a lower AP mae compared to the *sogD*_∆*Su*(*H*) by about 0.310 units with a *p*-value of 0.476. Based on this test, there was not a statistically significant difference in AP mae between the *sogD*_∆*Su*(*H*) and *sogD* groups.

However, the results for DV mae shows the *sogD* group had lower value by 1.620 units and 0.001 *p*-value. Also, the results for the mean mae indicates *sogD* had a lower value by 0.971 units and 0.019 *p*-value. The two latter results for DV mae and mean mae indicate significant difference between *sogD*_∆*Su*(*H*) and *sogD*.

**Figure 4:**
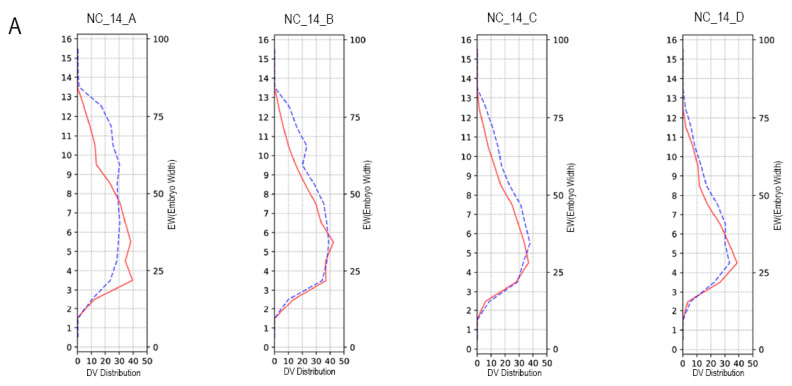

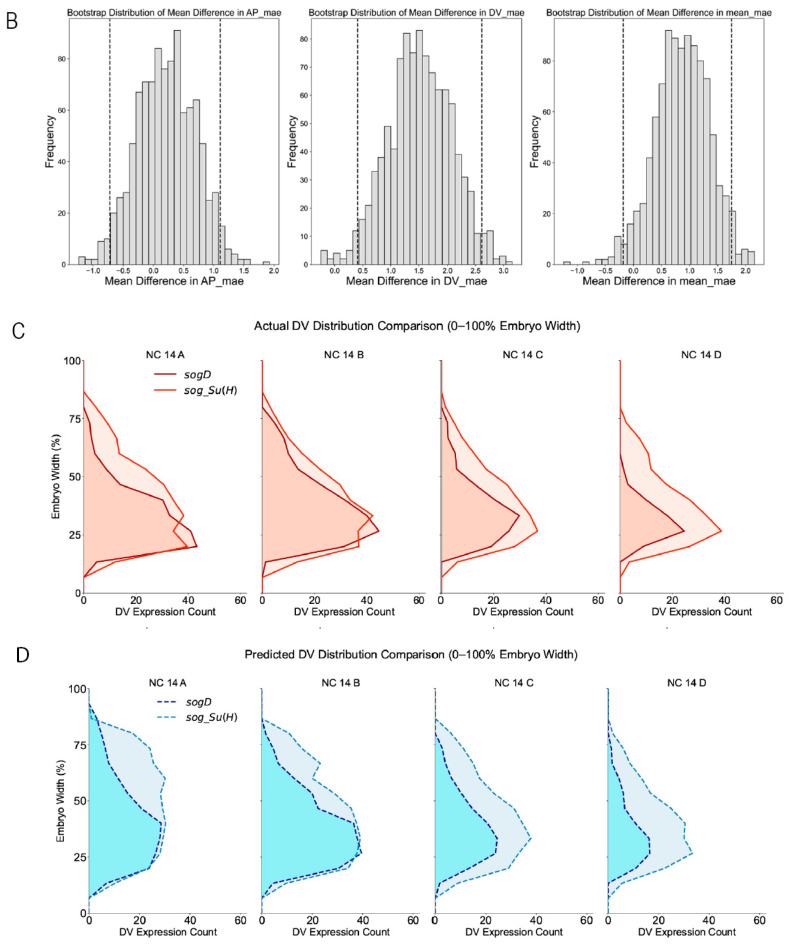
(**A**) Distribution of active cells along the DV axis for the *sogD*_∆*Su*(*H*) dataset, where the red line represents the actual distribution and the dashed blue line corresponds to the predicted distribution. (**B**) Bootstrap distribution results for *AP* → *mae*, *DV* → *mae*, and *mean*→*mae* presented from left to right, respectively. (**C**) Actual DV distribution for *sogD*_∆*Su*(*H*) and control datasets, shown in light and dark red, respectively, to illustrate changes in width over time. (**D**) Predicted DV distribution for *sogD*_∆*Su*(*H*) and control datasets, represented in dashed light and dark blue, respectively.

In addition, we implemented another empirical hypothesis testing called the Bootstrap method. Bootstrap methods can be used to estimate the distribution of our metrics under the null hypothesis. To implement the bootstrap, we used the same metrics as previous method. We drew samples from the original dataset with replacement to create a new dataset. Then, for each bootstrap sample, we computed the statistics of interest, which were DV mae, AP mae, and mean mae. By analyzing the bootstrap distribution, we found the confidence intervals for each metric. Figure 4B shows the bootstrap distribution of mean difference in AP mae, DV mae, and mean mae. It indicates that with a 95% confidence interval the mean difference in DV mae, (DV mae(*sogD_∆Su(H*)) − DV mae (*sogD)*) was between [0.409–2.61]. It can be concluded that, with a 95% confidence interval, the DV mae for *sogD_∆Su(H*) was at least 0.409 units higher than for *sogD*, which means the pipeline for *sogD* outperformed the *sogD_∆Su(H)* one. The ranges for AP mae and mean mae were, respectively, [−0.72–1.10] and [−0.18–1.74]. It can be seen that, for AP mae and mean mae, the ranges include zero, meaning the performance of *sogD* could be better, equal, or worse than *sogD_∆Su(H).* The result with the bootstrap method confirmed the results derived from the mixed-effects method, which makes sense given that large amounts of training data are needed to model transgenic effects. This indicates that the model more accurately predicted expression in the central region of both the wild-type and mutant *sogD* enhancers but showed reduced accuracy at the boundaries (Figure 4C,D).

## 3. Discussion

In this study, we aimed to explore the potential of machine learning in capturing the spatiotemporal dynamics of active nuclei during early *Drosophila* embryogenesis. Through a series of systematic ablation experiments, we optimized the model architecture, feature set, and grid size configuration to ensure robust predictions. Our pre-trained model was then evaluated on test data from two distinct datasets, *sogD*_∆*Su*(*H*) and *sogD*, which allowed us to assess its performance under different genetic contexts. Figure 3 highlights the model’s ability to accurately predict the distribution of active nuclei along the DV axis; the central focus of our study nicely shows the distribution and dynamic expression of *sog* gene at NC14.

One of the critical findings of this study was the model’s performance on the *sogD* dataset, as evidenced by the lower mean absolute error (mae) values along the DV axis. This suggests that the model is particularly adept at capturing the complex dynamics of active cell distributions under normal genetic conditions. However, the model also demonstrated its utility in detecting subtle differences between the *sogD*_∆*Su*(*H*) and *sogD* datasets, particularly in the stages NC13C and NC14D. In late NC14, a noticeable reduction in *sog*’s wild-type expression was observed. However, in the absence of the repressor Su(H), the distribution of active nuclei expanded across a much broader domain along the DV axis, highlighting the impact of the Su(H) mutation. This observation underscores the model’s sensitivity to changes in genetic background and further different experimental conditions.

Our comparison between the *sogD* and *sogD*_∆*Su*(*H*) datasets also provided valuable insights, validating its robustness in distinguishing between Su(H) in regulating the spatial distribution of active nuclei. The model’s ability to capture these nuanced differences offers promising potential for predicting the spatiotemporal dynamics of gene expression and cellular behaviors in various genetic contexts. Specifically, the results suggest that this approach could be extended beyond *sogD* to other DV expressed genes and genetic modifications, paving the way for broader applications in developmental biology.

In summary, our work makes several important contributions to the field of computational biology and developmental genetics. First, we developed an innovative and optimized imaging technology that provides super-resolution spatial information across the entire DV axis of the *Drosophila* embryo. Second, we introduced an automated, machine learning-driven pipeline that can accurately classify cell types based on their spatial characteristics. This is a significant step forward in overcoming the challenges of manual cell classification and provides a reliable tool for high-throughput analysis. Lastly, our model demonstrates the ability to predict the distribution of active cells at different developmental stages based on prior data, offering a powerful tool for modeling dynamic processes in embryonic development. In addition, these findings show that the predictive pipeline not only recapitulates known biological mechanisms but is also sensitive to subtle, context-dependent regulatory changes, underscoring its power as a tool for dissecting cis-regulatory logic. These advancements not only offer insights into the spatiotemporal regulation of DV genes but also hold promise for studying other genes and genetic mutations that influence embryogenesis.

An important direction for future work is to integrate mechanistic insights into transcriptional bursting as shown in previous work [14] with machine learning models that predict the large-scale dynamics of gene expression domains. Machine learning approaches can capture the global distribution of active nuclei across the dorsal–ventral axis and forecast changes in expression domains across developmental stages and genetic backgrounds. Published work demonstrates that Su(H) activity can exert opposite roles depending on spatial context, stabilizing bursts at the center of *sog* expression domain while restricting expression at the borders [14]. Similarly, this can be shown for a variety of broadly expressed transcription factors that exhibit a dual role in expression, functioning as both activators and repressors. Bridging these two perspectives would enable the development of models in which bursting parameters are incorporated as features in predictive pipelines. Such integration would allow models to forecast not only spatial expansion or restriction of gene expression domains but also underlying transcriptional dynamics, thereby extending predictions into later stages of embryogenesis. This approach allows for in silico enhancer mutagenesis, where the impact of specific binding site alterations on both local bursting behavior and expression patterns can be simulated prior to experimental validation, providing a powerful framework for understanding how cis-regulatory elements shape the transcriptional landscape during early embryogenesis.

## 4. Materials and Methods

### 4.1. ChIP-Seq and ATAC-Seq Analysis

Previously published ChIP-seq data were used to identify the binding sites of transcription factors and other chromatin-associated proteins in the *Drosophila* genome, providing insight on how these proteins regulate gene expression during embryogenesis. ChIP-seq libraries were obtained from the University of California, Santa Cruz (UCSC) Genome Browser. Reads from previous studies were aligned to the Drosophila reference genome assembly (UCSC dm3) [18,19,20]: Zelda at stage 5, Dorsal at stage 5, and Su(H) at stage 5. ATAC-seq data were used [15,21] to assess chromatin accessibility at the *sog* locus during NC14b in wild-type embryos as well as in mutants for the transcription factors Zelda and gd7 (nuclear Dorsal). ATAC-seq libraries were also obtained from the UCSC Genome Browser.

### 4.2. Experimental Set-Up for MS2.MCP Embryo Collection

Virgin females expressing MCP-GFP (green) and NupRFP (red) maternally were crossed with males carrying either the *sog Distal* eve2 promoter-MS2.yellow-attB or *sogD*_∆*Su*(*H*) eve2 promoter-MS2.yellow-attB [1,22]. This MS2 cassette contains 24 repeats of a DNA sequence that produces an RNA stem loop when transcribed [23]. The stem loop structure is specifically bound by the phage MS2 coat protein (MCP). MCP fused to GFP binds to MS2-containing transcripts (i.e., *sog Distal*.MS2), producing a strong green signal within the nuclei of *Drosophila* embryos at sites of nascent transcript production [24,25]. Embryos were precisely timed and collected during nuclear cycles 10–11.

### 4.3. Live Imaging

In this MS2.MCP system, nuclear GFP fluorescence was observed as a single dot per nucleus in heterozygous individuals, corresponding to nascent transcription from a single copy of the MS2-containing reporter transgene integrated into the genome [26]. Furthermore, the nuclear periphery was marked by a fusion of RFP to nuclear pore protein (Nup-RFP) [27]. We optimized the imaging protocol to provide spatial information across the entire dorsal–ventral (DV) axis of embryos with the fastest temporal resolution that also retains embryo viability. In brief, embryos were imaged on a Zeiss LSM 900 continuously over the course of 2 h at an interval of 30 s per scan (twice as fast compared to previous studies). Importantly, this imaging protocol was not phototoxic to embryos. Embryos were collected on apple agar plates for 1 h, rested for 30 min at room temperature, and manually dechorionated. They were mounted between a slide and coverslip using heptane-dissolved adhesive and immersed in Halocarbon 27 oil. Imaging was performed on a Zeiss LSM 900 Airyscan 2 (Zeiss, Oberkochen, Germany) during stages leading into gastrulation, with broad-view and super-resolution movies captured using a 40× water oil immersion objective. Images were acquired at varying resolutions and intervals, as described [14]. For imaging analysis and machine learning processing of gene expression dynamics, all frames of the resulting 2D movies were divided into separate stages by nuclear cycle, including four sub-stages for NC14 (NC14A, NC14B, NC14C, and NC14D) [14].

### 4.4. Data Preprocessing

**Figure 5:**
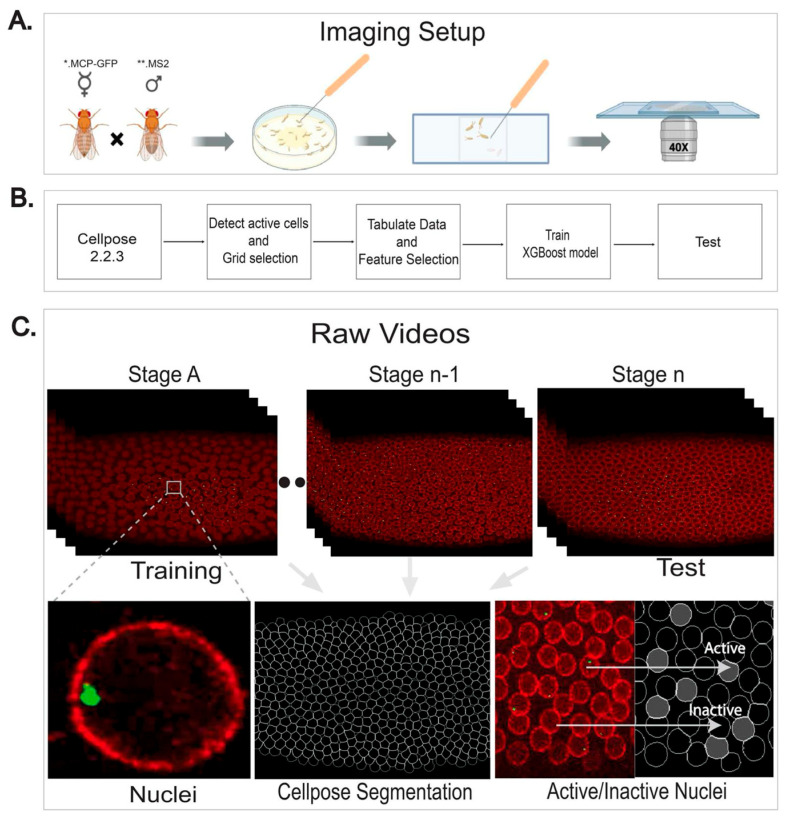
Preprocessing data model. Computational analysis of super-resolution live imaging compares nuclei activity and predicts gene expression outputs. (**A**) Super-resolution live imaging set-up of hand-dechorionated *Drosophila* embryos of Nup-RFP (*) MCP-GFP (*.MCP-GFP) X *sogD*_∆*Su*(*H*).MS2. The symbol “**” denotes *sogD or sogD*_∆*Su*(*H*) constructs (**.MS2) (**B**) Implemented pipeline, starting with using Cellpose 2.2.3 for segmentation, followed by subsequent stages involving active nuclei detection, tabulating data and feature selection, training, and testing. These steps collectively aimed to predict the distribution of active cells for the next stage. (**C**) The MS2.MCP-GFP system tracked transcription via GFP-*tagged* MCP binding to MS2 loops (Stage A-NC13, double-dot “.” NC14A, NC14B, Stage n-1 = NC14C, Stage n = NC14D) and nuclei activity of live imaging snapshots was compared with Cellpose-generated images.

We conducted preprocessing, feature extraction, training, and testing, as shown in Figure 5. Training and testing followed the same preprocessing and feature extraction steps. The videos show real time images from embryonic development, which were manually given stage development labels: NC13, NC14A, NC14B, NC14C, and NC14D. In the preprocessing step, we used a generalist, deep learning-based segmentation method called Cellpose, which could precisely segment cells in each frame of embryo development. Active cells were identified based on prevalence of green pixels, which were indicative of gene expression within the cell, and the active mask underwent feature extraction. During this stage, the masked images underwent a gridding procedure with a predetermined size.

Subsequently, the entire imaging dataset was transformed into a tabular format, considering the spatial information of each cell. We utilized four different metrics to capture both local and global features in a frame including m1, m2 for both AP and DV axes, Ripley’s K-function, and *n* (total number of cells in each grid). Here, m1 and m2 denote the first and second moments, respectively, capturing the distribution of active cells at each stage. Furthermore, Ripley’s K-function was employed to analyze spatial correlation and quantify deviations from a random spatial distribution. Equation (1) illustrates the formula for calculating Ripley’s K-function (the same method was proposed to capture spatial proteomics data to map cell states for cancer patient survival prediction). Where *A* is the area under each window with constant radius, *n* is the number of data points, *d_ij_* is the distance between two points, and *e_ij_* is an edge correction weight. Then, the tabular data went through two steps of averaging on each stage and time correcting. Since our goal was to predict the distribution of active cells in each stage and we had different numbers of frames for each stage, we averaged the whole feature values based on each stage. Also, to account for temporal alignment, we implemented a one-stage shift in features, where we utilized the features from the previous stage in prediction of the current stage.

(1) $$\;{\hat{K}}_{r}=\frac{A}{n\left(n-1\right)}\sum_{i=1\;}^{n}\sum_{j=1,j\neq i\;}^{n}1\left(d_{ij}\;\leq \;r\right)e_{ij}$$

### 4.5. Training

Following the completion of the feature extraction process, the dataset underwent preparation for training an XGBoost model, a supervised learning algorithm. The outcome of this pipeline was the count of active cells within each grid at a given stage, determined by the features from the preceding stage.

### 4.6. Evaluation

Subsequent to training the model, its performance was evaluated using test data. During testing, all pre-processing and feature extraction steps were replicated, and the pre-trained XGBoost model was employed to forecast the count of active cells for each grid across various stages.

## Figures and Tables

**Table 1:** The average mae value on K-fold cross validation over test dataset for different combinations of features for ablation study.

Feature List	Mae
*n*, Ripley’s K-function	3.799
m2, *n*, Ripley’s K-function	3.86
m2, m1 AP, *n*, Ripley’s K-function	3.92
Ripley’s K-function	3.93
m2, m1 AP, m1 DV, Ripley’s K-function	3.94

## Data Availability

Data is contained within the article.
